# 15-Lipoxygenase metabolites of α-linolenic acid, [13-(S)-HPOTrE and 13-(S)-HOTrE], mediate anti-inflammatory effects by inactivating NLRP3 inflammasome

**DOI:** 10.1038/srep31649

**Published:** 2016-08-18

**Authors:** Naresh Kumar, Geetika Gupta, Kotha Anilkumar, Naireen Fatima, Roy Karnati, Gorla Venkateswara Reddy, Priyanka Voori Giri, Pallu Reddanna

**Affiliations:** 1 School of Life Sciences, University of Hyderabad, Hyderabad 500046, India; 2 National Institute of Animal Biotechnology, Hyderabad 500049, India

## Abstract

The ratio of ω-6 to ω-3 polyunsaturated fatty acids (PUFAs) appears to be critical in the regulation of various pathophysiological processes and to maintain cellular homeostasis. While a high proportion of dietary intake of ω-6 PUFAs is associated with various inflammatory disorders, higher intake of ω-3 PUFAs is known to offer protection. It is now well established that beneficial effects of ω-3 PUFAs are mediated in part by their oxygenated metabolites mainly via the lipoxygenase (LOX) and cyclooxygenase (COX) pathways. However, the down-stream signaling pathways that are involved in these anti-inflammatory effects of ω-3 PUFAs have not been elucidated. The present study evaluates the effects of 15-LOX metabolites of α-linolenic acid (ALA, ω-3 PUFA) on lipopolysaccharide (LPS) induced inflammation in RAW 264.7 cells and peritoneal macrophages. Further, the effect of these metabolites on the survival of BALB/c mice in LPS mediated septic shock and also polymicrobial sepsis in Cecal Ligation and Puncture (CLP) mouse model was studied. These studies reveal the anti-inflammatory effects of 13-(S)-hydroperoxyoctadecatrienoic acid [13-(S)-HPOTrE] and 13-(S)-hydroxyoctadecatrienoic acid [13-(S)-HOTrE] by inactivating NLRP3 inflammasome complex through the PPAR-γ pathway. Additionally, both metabolites also deactivated autophagy and induced apoptosis. In mediating all these effects 13-(S)-HPOTrE was more potent than 13-(S)-HOTrE.

Inflammation is an essential part of host’s response to infection or injury in order to maintain cellular homeostasis. Aberrant inflammation is associated with various disorders mediated by hyperactivation of inflammasome complexes and up-regulation of pro-inflammatory enzymes like inducible nitric oxide synthase (iNOS)^1^,^2^, LOX^3^ and COX^4^,^5^. The inflammatory response generated by eicosanoids, the oxygenated metabolites of PUFAs such as arachidonic acid (AA) is known to be mediated by pro-inflammatory cytokines such as IL-1β and tumor necrosis factor-α (TNF-α)^6^.

Increased ratio of ω-6 to ω-3 PUFAs is hypothesized to elevate pro-inflammatory eicosanoid production and thus the onset of inflammatory diseases. A sufficiently high intake of ω-3 PUFAs, on the other hand, was shown to offer protection from inflammatory diseases by decreasing the production of pro-inflammatory eicosanoids, cytokines, ROS and RNS^7^,^8^. In addition, it is reported that increasing ω-3 PUFAs tissue levels by dietary or genetic means decrease the pathological retinal angiogenesis by suppressing TNF-α^9^. These beneficial effects of ω-3 PUFAs appear to be mediated by the oxygenated metabolites formed via the LOX and COX pathways^10^,^11^,^12^,^13^,^14^.

COX-2, an inducible enzyme that converts ω-6 PUFAs such as AA to pro-inflammatory prostaglandins, has been widely recognized as the major pathway responsible for inflammation as it triggers the production of other pro-inflammatory chemokines and cytokines^14^. However, this concept is challenged by recent findings on COX-2 derived oxidative metabolites of ω-3 PUFAs possessing anti-inflammatory and anti-oxidant properties^15^,^16^. These studies suggest that the ultimate pro/anti-inflammatory effects of COX depend on the substrate on which these enzymes act, ω-6 or ω-3 PUFAs^17^,^18^ and their down-stream metabolites-PGE_2_ and/or PGD_2._ While PGE_2_ is generally pro-inflammatory in nature^13^, PGD_2_ exhibits anti-inflammatory effects by its conversion to PGJ_2_ and subsequently 15-deoxy-Δ^12^,^14^ PGJ_2_ (15d-PGJ_2_) a well-known ligand with high affinity for peroxisome proliferator-activated receptor-γ (PPAR-γ)^19^,^20^,^21^. LOXs, on the other hand, are majorly classified into 5-, 12- and 15-LOXs, depending on the position at which AA is oxygenated. These LOX isoforms have been implicated in a variety of inflammatory and hyperproliferative and neurodegenerative diseases^22^. While 5-LOX is pro-inflammatory in nature^23^, the 15-LOX exhibits anti-inflammatory properties^24^,^25^. Earlier we have shown the anti-inflammatory and anti-cancer properties of 15-LOX metabolites of AA and elucidated the mechanisms involved^26^,^27^,^28^. In the present study, we report the anti-inflammatory effects of 15-LOX metabolites of ALA, the precursor for eicosapentaenoic acid (EPA) and docosahexaenoic acid (DHA), on LPS stimulated mouse macrophage cell line, RAW 264.7 and primary peritoneal macrophages isolated from BALB/c mice and demonstrated that these effects are mediated by inactivating NLRP3 inflammasome complex through the PPAR-γ pathway. Further, we report on the extended survival of BALB/c mice in endotoxin-mediated septic shock and polymicrobial sepsis in CLP mouse model.

## Results

### Hydroperoxy metabolites exhibit more cytotoxic effects on RAW 264.7 cells as compared to hydroxy metabolites of ALA

**Figure 1:**
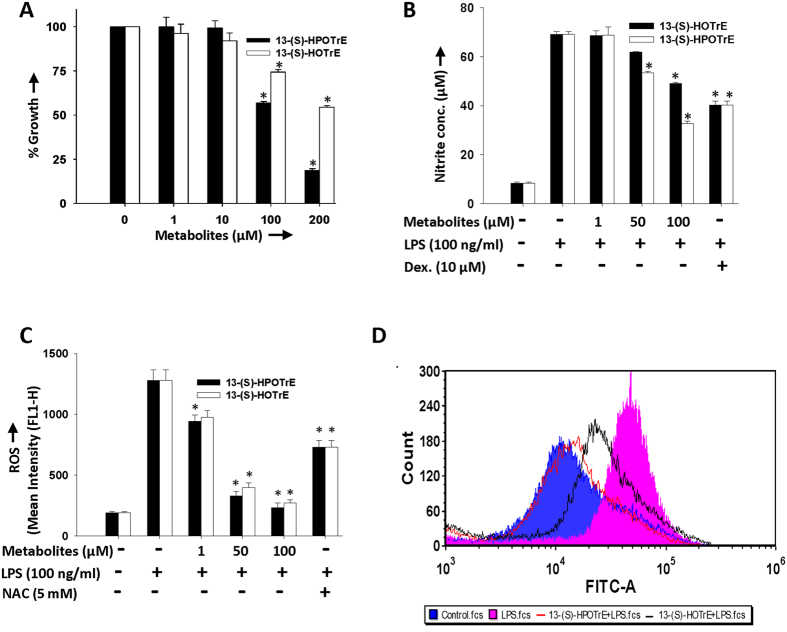
Effects of 13-(S)-HPOTrE and 13-(S)-HOTrE on cells viability, generation of NO and ROS in RAW 264.7 cells and mouse peritoneal macrophages. (**A**) Effects of 13-(S)-HPOTrE and 13-(S)-HOTrE on the viability of RAW 264.7 cells were measured by MTT assay. Cells were treated with different concentrations (1, 10, 100 and 200 μM) of ALA metabolites for 24 h. The percent cell growth following treatment was calculated, in comparison with untreated control cells. The values represent mean ± SD of three independent experiments. *Indicates significance (p < 0.05) when compared with untreated control cells. (**B**) Nitrite levels in the culture medium of RAW 264.7 cells pre-incubated with different concentrations (1, 50 and 100 μM) of ALA metabolites or dexamethasone (10 μM) for 3 h and further stimulated with or without LPS (100 ng/ml) for the next 24 h. 13-(S)-HPOTrE showed more effective dose-dependent reduction in NO level as compared to 13-(S)-HOTrE. Dexamethasone, a steroidal anti-inflammatory drug that suppresses NO production, is used as positive control. *Indicates significance (p < 0.05) compared to LPS treated cells. (**C**) Intracellular ROS level in RAW 264.7 cells following pretreatment with 100 μM of ALA metabolites for 3 h then stimulated with LPS for 16 h. 13-(S)-HPOTrE reduced ROS level more efficiently compared to 13-(S)-HOTrE. N-Acetyl Cysteine (NAC, 5 mM), a ROS inhibitor was used as positive control. The values represent mean ± SD of three independent experiments. *Indicates significance (p < 0.05) compared to LPS treated cells. (**D**) Intracellular ROS level in peritoneal macrophages following pre-incubation with 100 nM ALA metabolites for 3 h then challenged with LPS for 16 h. Similar to above result, 13-(S)-HPOTrE reduced ROS level more efficiently compared to 13-(S)-HOTrE. This is representative FACS chromatogram of the three independent experiments.

The cytotoxic effects of 15-LOX metabolites of ALA [13-(S)-HPOTrE and 13-(S)-HOTrE] on RAW 264.7 cells were evaluated by MTT (3-(4, 5-dimethylthiazol-2-yl)-2, 5-diphenyl tetrazolium bromide) assay. Among this, hydroperoxy metabolite, 13-(S)-HPOTrE showed more cytotoxic effect compared to the hydroxy metabolite, 13-(S)-HOTrE, when incubated at different concentrations for 24 h (Fig. 1A). Treatment with both metabolites decreased the proliferation of the cells in a dose and time-dependent manner. However, 13-(S)-HPOTrE and 13-(S)-HOTrE showed more cytotoxic effects when compared to ω-3 (ALA & DHA) and ω-6 (AA & Linoleic acid-LA) PUFAs (Supplementary Figure S1). A 50% decrease in RAW 264.7 cell proliferation was observed at a concentration (IC_50_ value) of 114 μM 13-(S)-HPOTrE, which was much lower than 13-(S)-HOTrE (>200 μM) at 24 h. Based on these results, further experiments on RAW 264.7 cells were carried out up to 100 μM concentration of ALA metabolites for analysis of their anti-inflammatory effects.

### ALA metabolites reduce nitric oxide levels and ROS generation in RAW 264.7 cells and peritoneal macrophages

Next, it was aimed to evaluate the effects of ALA metabolites on LPS induced inflammation by examining effects on pro-inflammatory parameters: NO- a second messenger and ROS- an oxidative stress marker. For this, RAW 264.7 cells were first treated with and/or without metabolites and challenged with LPS as described in the methods. There was significant elevation in NO production and ROS generation in cells when stimulated with LPS and this elevated NO level was reduced by 52.7% and 29% in the presence of 13-(S)-HPOTrE and 13-(S)-HOTrE at 100 μM, respectively (Fig. 1B). Similarly, LPS stimulated RAW 264.7 cells, pre-treated with 13-(S)-HPOTrE and 13-(S)-HOTrE, resulted in the reduction of ROS generation, in a dose-dependent manner and at 100 μM concentration, decreased ROS generation by 96.4% and 92.7% respectively (Fig. 1C). Not only ALA metabolites, the substrate fatty acid ALA also showed the similar pattern of decreased ROS and NO production in LPS stimulated RAW 264.7 cells, though lower (18.6% and 89.5%) compared to its metabolites (Supplementary Figure S2). Similar effects were observed on ROS level when the mouse peritoneal macrophages were pre-treated with ALA metabolites at lower doses (100 nM) and then challenged with LPS (Fig. 1D). The significant reduction in the production of the pro-inflammatory markers by the ALA metabolites suggest their strong anti-inflammatory effects on LPS stimulated macrophages.

### ALA metabolites inhibit the expression of iNOS and TNF-α in LPS stimulated RAW 264.7 cells

**Figure 2:**
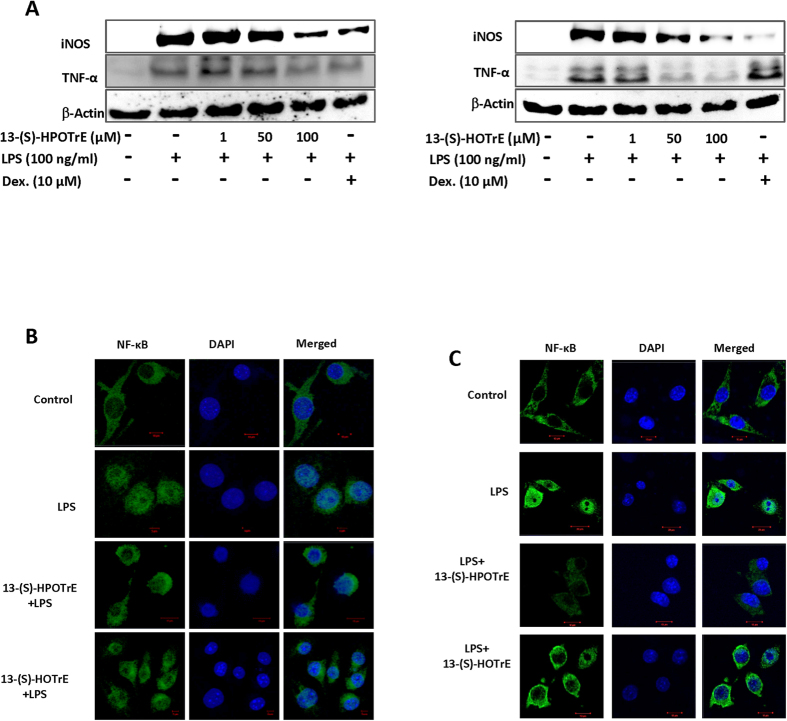
Anti-inflammatory effect of 13-(S)-HPOTrE and 13-(S)-HOTrE is mediated by inhibition of iNOS and TNF-α. (**A**) Immunoblot analysis showing the expression of iNOS and TNF-α following treatment with 13-(S)-HPOTrE and 13-(S)-HOTrE (1, 50 and 100 μM) concentrations for 3 h and then stimulated with LPS for 24 h. β-Actin was used as an internal control. Dexamethasone (10 μM) was used as positive control. These are representative blots of the three independent experiments. (**B**) Immunofluorescence microscopy of RAW 264.7 cells pre-treated with or without ALA metabolites (100 μM) for 3 h and then with LPS for 16 h. Immunostained for NF-κB (Green) and DAPI (Blue). Bar Scale: 10 μm. The image showed representative of three independent experiments. (**C**) Immunofluorescence microscopy of peritoneal macrophages pre-treated with or without ALA metabolites (100 nM) for 3 h and then challenged with LPS for 16 h. Immunostained for NF-κB (Green) and DAPI (Blue), Bar Scale: 10 μm. The image showed representative of three independent experiments.

iNOS is an enzyme associated with regulation of NO and ROS generation in monocytes, macrophages, and other cells^29^. The expression of iNOS and TNF-α, in RAW 264.7 cells stimulated with LPS in the presence and/or absence of ALA metabolites was studied. 13-(S)-HPOTrE or 13-(S)-HOTrE showed no effect on the expressions of either iNOS or TNF-α as compared to untreated RAW 264.7 cells (Supplementary Figure S3), while LPS induced their expression. However, their expression in RAW 264.7 cells, pre-treated with 13-(S)-HPOTrE and 13-(S)-HOTrE and then challenged with LPS, was reduced in a dose-dependent manner (Fig. 2A). The transcript levels of iNOS and TNF-α were also analyzed by semi-quantitative PCR. In LPS treated RAW 264.7 cells, the transcript levels of iNOS and TNF-α were increased but were reduced by pre-treatment with 13-(S)-HPOTrE and 13-(S)-HOTrE in a dose-dependent manner (Supplementary Figure S4). NF-κB is known to regulate the expression of various pro-inflammatory genes, including iNOS and TNF-α^30^,^31^ and ALA metabolites may affect its translocation to the nucleus to regulate gene expression. Therefore the activation of NF-κB was monitored for its translocation to the nucleus by confocal microscopy. While LPS stimulation of RAW 264.7 cells showed a marked translocation of NF-κB, 13-(S)-HPOTrE and 13-(S)-HOTrE pre-treatments resulted in a decrease in NF-κB translocation (Fig. 2B). Very similar effects were observed on NF-κB translocation when the mouse peritoneal macrophages were treated with 13-(S)-HPOTrE and 13-(S)-HOTrE at a lower dose (100 nM) and then challenged with LPS (Fig. 2C).

### 13-(S)-HPOTrE and 13-(S)-HOTrE inactivate inflammasome in PPAR-γ dependent manner in RAW 264.7 cells and peritoneal macrophages

**Figure 3:**
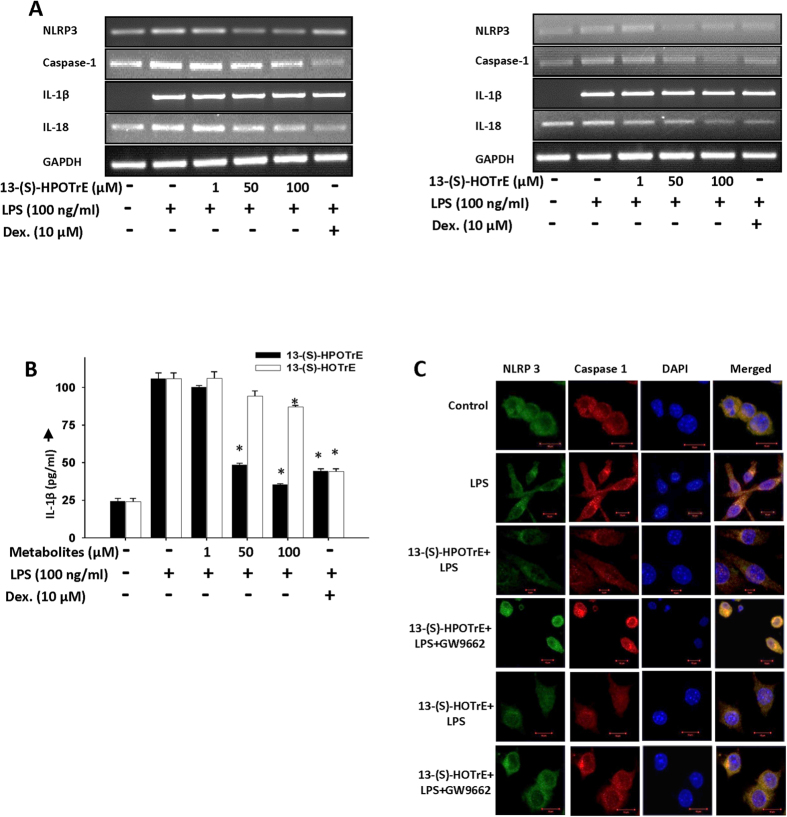
ALA metabolites, 13-(S)-HPOTrE and 13-(S)-HOTrE, inhibit inflammasome complex in RAW 264.7 cells. (**A**) In semi-quantitative PCR analysis, 13-(S)-HPOTrE, and 13-(S)-HOTrE showed decrease in transcript level of NLRP3, caspase-1, IL-1β and IL-18 at various concentrations (1, 50 and 100 μM) pre-incubated for 3 h then stimulated with LPS for 24 h. GAPDH was used as an internal control. (**B**) Estimation of IL-1β levels in the culture medium of cells as per the treatment described in (**A**). Dexamethasone (10 μM) was used as positive control. The values represent mean ± SD of three independent experiments. *Indicates significance (p < 0.05) compared to LPS alone treated cells. (**C**) Immunofluorescence microscopy of RAW 264.7 cells pre-treated with ALA metabolites (100 μM) for 3 h then stimulated with LPS for 24 h. Immunostained for NLRP3 (Green), caspase-1 (Red) and DAPI (Blue). Bars Scale: 10 μm. Data show representative of three independent experiments.

It is well known that NLRP3 inflammasome, an intracellular sensor that detects pathogens and sterile inflammation, gets activated in part through NF-κB in response to LPS treatment^32^,^33^,^34^. For analyzing the effects of 15-LOX metabolites of ALA on NLRP3 inflammasome activation in LPS stimulated RAW 264.7 cells, the level of gene transcripts associated with inflammasome complex were quantified by semi-quantitative PCR. An increased transcript level of NLRP3, caspase-1, IL-1β and IL-18 in LPS activated RAW 264.7 cells showed an efficient dose-dependent reduction on treatment with both 13-(S)-HPOTrE and 13-(S)-HOTrE (Fig. 3A). Since activated inflammasomes initiate the processing and secretion of IL-1β^35^,^36^, we measured the level of IL-1β, in the culture medium of RAW 264.7 cells by ELISA. These studies showed a 4.4 fold increase in the level of IL-1β in response to LPS treatment in RAW 264.7 cells and this elevated level of IL-1β was significantly reduced by the treatment of 13-(S)-HPOTrE and 13-(S)-HOTrE, in a dose-dependent manner (Fig. 3B). At 100 μM concentration, 13-(S)-HPOTrE and 13-(S)-HOTrE reduced IL-1β levels by 86.4% and 23% respectively.

LOX metabolites are natural ligands of PPAR-γ^37^,^38^,^39^,^40^. Interestingly, PPAR-γ and their coactivators were shown to promote macrophage’s anti-inflammatory properties to increase insulin sensitivity^41^. In the present scenario, we studied the involvement of PPAR-γ on the NLRP3 inflammasome activation in LPS treated RAW 264.7 cells and peritoneal macrophages. Immunofluorescence microscopy studies showed activation of NLRP3 inflammasome complex along with upregulation of caspase-l upon LPS treatment in RAW 264.7 cells (Fig. 3C). However, pre-treatment of RAW 264.7 cells with 13-(S)-HPOTrE or 13-(S)-HOTrE and then challenged with LPS resulted in inactivation of NLRP3 inflammasome and thus downregulation of caspase-1. Interestingly, these effects of 13-(S)-HPOTrE and 13-(S)-HOTrE on inflammasome and caspase-1 were reversed when co-incubated with GW9662, a PPAR-γ antagonist.

**Figure 4:**
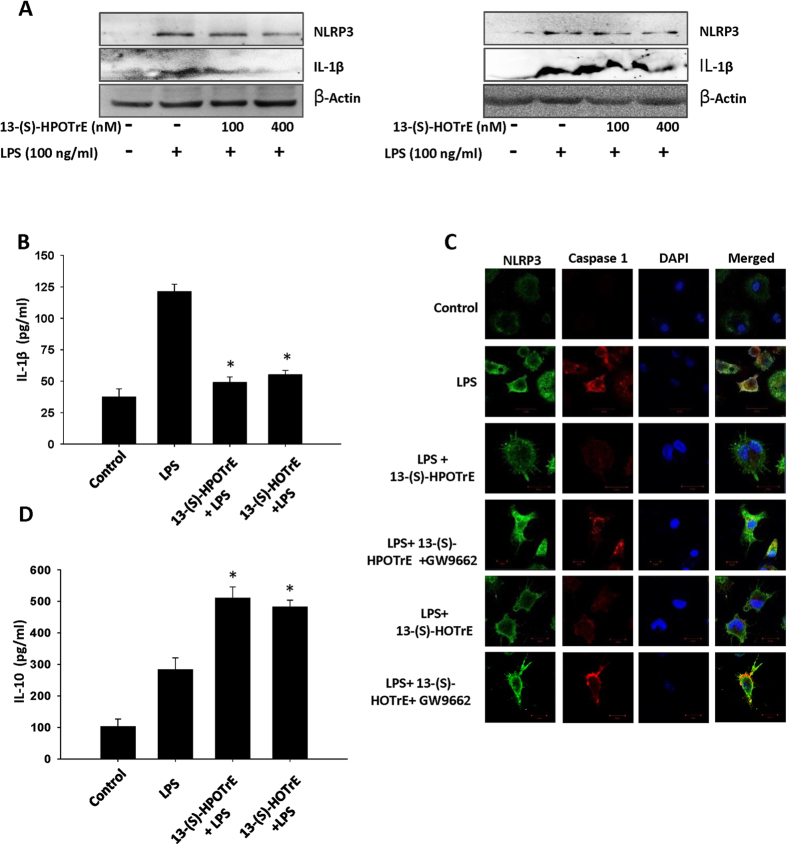
ALA metabolites inhibit inflammasome complex activation in mouse peritoneal macrophages. (**A**) Immunoblot analysis of NLRP3 and IL-1β (in culture medium) expression in peritoneal macrophages, pre-incubated with ALA metabolites (100 nM and 400 nM) for 3 h then challenged with LPS (100 ng/ml) for next 24 h. β-Actin was used as an internal control. (**B**) Estimation of IL-1β levels in the culture medium by ELISA as per the treatment described above. The values represent mean ± SD of three independent experiments. *Indicates significance (p < 0.05) compared to LPS treated peritoneal macrophages. (**C**) Immunofluorescence microscopy of peritoneal macrophages treated with ALA metabolites (100 nM) for 3 h then stimulated with LPS for 24 h. Immunostained for NLRP3 (Green), caspase-1 (Red) and DAPI (Blue). Bars Scale: 10 μm. Images show representative of three independent experiments. (**D**) Estimation of IL-10 levels in the culture medium by ELISA as per the treatment described above (at 100 nM). The values represent mean ± SD of three independent experiments. *Indicates significance (p < 0.05) compared to LPS treated peritoneal macrophages.

To validate the findings observed in RAW 264.7 cells, further, studies were taken up on peritoneal macrophages at much lower doses (100 nM and 400 nM) of 13-(S)-HPOTrE and 13-(S)-HOTrE. Immunoblot analysis showed an increase in the expression of NLRP3 in LPS challenged peritoneal macrophages while cells pre-treated with ALA metabolites and challenged with LPS showed a reduction in the expression of NLPR3 (Fig. 4A). Next, the IL-1β cytokine was probed in the culture medium by immunoblot analysis and quantified by ELISA. As shown in Fig. 4A,B, peritoneal macrophages pre-treated with 13-(S)-HPOTrE and 13-(S)-HOTrE and then challenged with LPS reduced the level of IL-1β by 59.5% and 54.5% respectively, when compared to LPS alone challenged macrophages. Similarly, the involvement of PPAR-γ in the regulation of inflammasome by ALA metabolites was validated on peritoneal macrophages at lower concentrations. Immunofluorescent microscopy showed decreased the expression of NLRP3 inflammasome and caspase-1 in macrophages pre-treated with ALA metabolites and challenged with LPS. However, the effects of ALA metabolites were reversed when co-incubated with GW9662, PPAR-γ antagonist (Fig. 4C). These metabolites also increased the levels of anti-inflammatory cytokine-IL-10 in culture medium (Fig. 4D).

**Figure 5:**
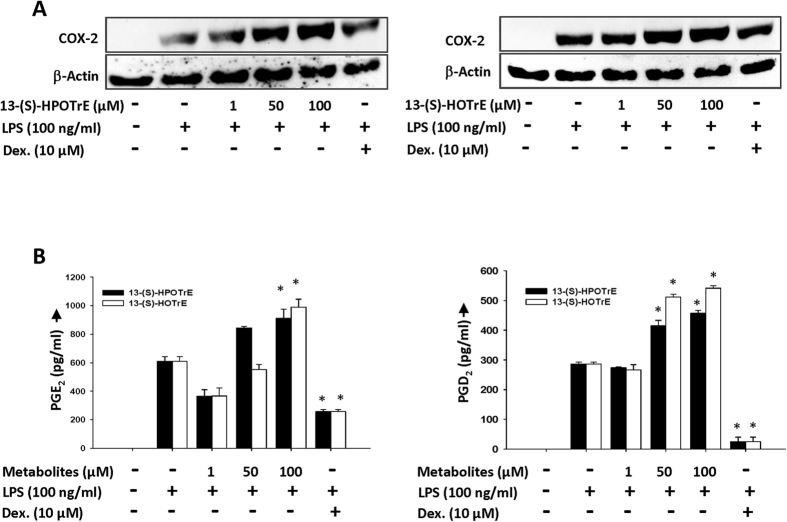
13-(S)-HPOTrE and 13-(S)-HOTrE show anti-inflammatory effects by upregulating expression of COX-2. (**A**) Immunoblot analysis of COX-2 expression in RAW 264.7 cells pre-incubated with ALA metabolites at different concentrations (1, 50 and 100 μM) for 3 h then further stimulated by LPS (100 ng/ml) for next 24 h. β-Actin was used as an internal control and Dexamethasone was used as positive control. Western blot shows representative of three independent experiments. (**B**) Estimation of PGE_2_ and PGD_2_ level in the culture medium of RAW 264.7 cells when pre-incubated with ALA metabolites for 3 h and then stimulated with LPS for 24 h. Dexamethasone was used as positive control. The values represent mean ± SD of three independent experiments. *Indicates significance (p < 0.05) compared to LPS treated cells.

The foregoing results suggest that inactivation of the inflammasome by 13-(S)-HPOTrE and 13-(S)-HOTrE in LPS challenged RAW 264.7 cells and peritoneal macrophages is mediated through PPAR-γ dependent pathway. Immunoblot analysis of the LPS stimulated RAW 264.7 cells revealed an increase in the COX-2 expression (Fig. 5A). The LPS induced expression of COX-2 was further enhanced by both 13-(S)-HPOTrE and 13-(S)-HOTrE in a dose-dependent manner. Coinciding with the changes observed in COX-2, the downstream metabolites of COX-2, PGE_2_ and PGD_2,_ also showed similar trend of enhanced production with LPS treatment (Fig. 5B). These studies reveal that COX-2, a key enzyme involved in mediating inflammation, may also be playing a role in the resolution of inflammation through the generation of PGD_2_ which is a PPAR-γ ligand^17^.

### ALA metabolites induce apoptosis in LPS treated RAW 264.7 cells

**Figure 6:**
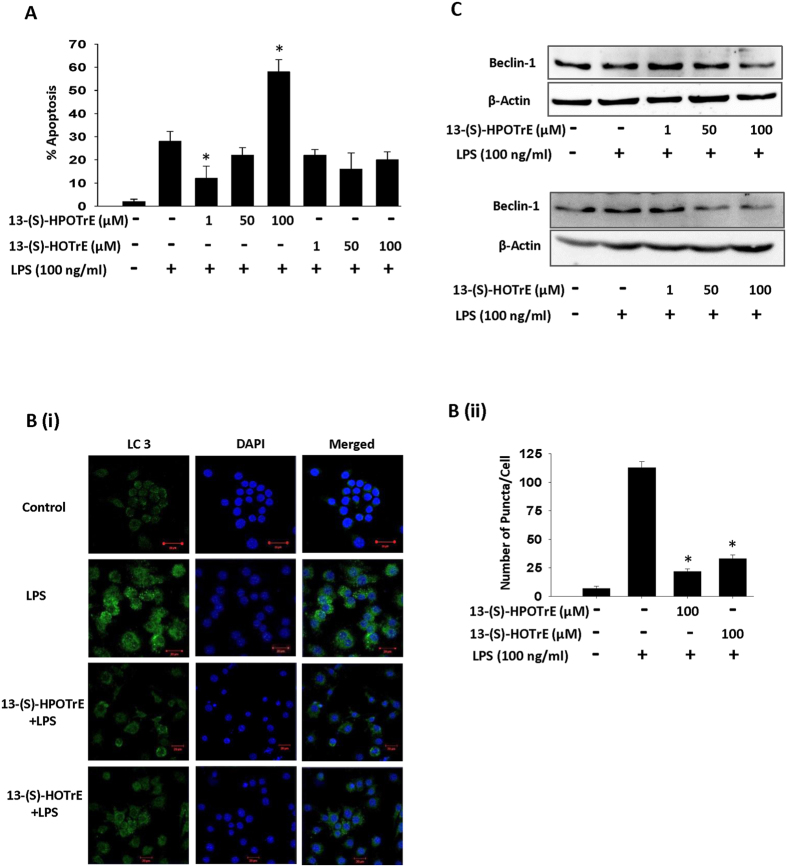
13-(S)-HPOTrE and 13-(S)-HOTrE inhibit Beclin-1 mediated autophagy in LPS stimulated RAW 264.7 cells. (**A**) Flow cytometric analysis showing the effects of ALA metabolites on apoptosis in RAW 264.7 cells challenged with LPS. Apoptosis was assayed by Propidium Iodide and FITC conjugated Annexin V at 24 h, by flow cytometric Analysis. The apoptosis level was calculated as % of AnnexinV^+^ PI^**−**^ cells in density plot distribution. The dead cells were gated and analysis was performed only on live cells. *Indicates significance (p < 0.05) compared to LPS treated cells. (**B**) **(i)** Confocal microscopy of RAW 264.7 cells treated with or without ALA metabolites (100 μM) for 3 h then challenged with LPS for 24 h. Immunostained for LC 3 (Green) and DAPI (Blue). Bars Scale: 10 μm. Images show representative of three experiments. (**ii)** Bar graph shows puncta/cell calculated by ImageJ LC3 macro. *Indicates significance (p < 0.05) compared to LPS treated cells. (**C**) Beclin-1 Immunoblotting of RAW 264.7 cells pre-incubated with ALA metabolites for 3 h and then challenged with LPS for 24 h. Western blots show representative of three independent experiments.

It is known that apoptosis plays a vital role in the resolution of inflammation and Beclin-1 dependent inhibition of autophagy by apoptosis enhances its anti-inflammatory effects^42^. Apoptosis blocks Beclin-1 dependent autophagy by blocking autophagosome synthesis^43^. Moreover, LPS and activated inflammasomes also induce autophagy in mesothelial cells^44^. Since ALA metabolites showed anti-inflammatory effects, we examined whether they affect induction of apoptosis and autophagy in LPS stimulated RAW 264.7 cells. 13-(S)-HPOTrE treatment increased apoptosis in LPS challenged RAW 264.7 cells in a dose-dependent manner (Fig. 6A) although, 13-(S)-HPOTrE and 13-(S)-HOTrE showed no effect on apoptosis in unchallenged cells (Supplementary Figure S5). A significant increase in autophagy with the enhanced conversion of soluble LC3-I into lipid bound LC3-II and increased puncta formation was observed in LPS challenged RAW 264.7 cells. However, these effects were greatly reduced or undetectable in RAW 264.7 cells pre-treated with ALA metabolites and challenged with LPS (Fig. 6B). Moreover, immunoblot analysis also showed induced expression of beclin-1, an autophagic marker, which was greatly reduced when RAW 264.7 cells were treated with ALA metabolites in a dose-dependent manner (Fig. 6C).

### ALA metabolites extend BALB/c mice survival and show anti-inflammatory properties by regulating cytokines and inflammasome

**Figure 7:**
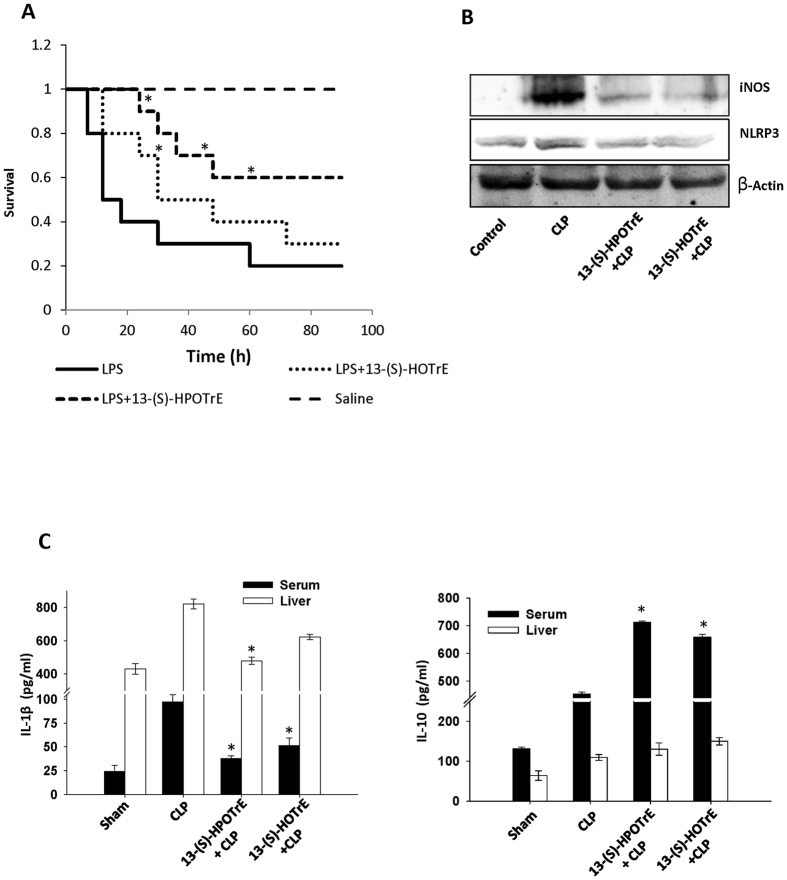
Effects of ALA metabolites during sepsis in BALB/c mice. (**A**) ALA metabolites showed an increase in survival of BALB/c mice (n = 10 mice/group) in endotoxin-induced Septic Shock in toxemia model. LPS (30 mg/kg) was injected i.p. and mice were monitored for 4 days then mortality was calculated. ALA metabolites showed decreased mortality rate in metabolites treated and LPS challenged mice as compared to LPS challenged mice. The survival curve is plotted on Kaplan-Meier method. *Indicates significance (p < 0.05) compared to LPS treated mice. (**B**) Sepsis was induced by polymicrobial infection in CLP mouse model (n = 5 mice/group). Immunoblot analysis of iNOS and NLRP3 expression in CLP mice model, pre-treated with 13-(S)-HPOTrE and 13-(S)-HOTrE (0.1 mg/kg, 2.5 μg/mice) as described in methodology. Mice were sacrificed after 24 h of CLP and tissues were collected. β-Actin was used as an internal control. (**C)** Estimation of IL-1β and IL-10 cytokines levels in serum and liver tissue. The values represent mean ± SD of three independent experiments. *Indicates significance (p < 0.05) compared to CLP mice.

Septic shock is a complication of inflammation in which an infection or toxin induces inflammatory response in the entire body. Endotoxin-mediated septic shock is often integrated with high mortality^45^. On the other hand, saturated fatty acid had shown major impact on the survival of mice in bacterial infection^46^ and these ALA metabolites showed anti-inflammatory effects in *in vitro* model system. We then wanted to validate the effects of ALA metabolites *in vivo* on the survival of mice in a septic shock induced by LPS in toxemia model. These studies showed features of septic shock in all the mice received LPS such as an increase in heartbeat rate, decrease in body weight, apathy, dullness and diarrhea, which began to die within 12 h. Contrary to this, the mice pre-treated with ALA metabolites and then challenged with LPS were quite active. However, loss of weight was observed in all except control mice. The result from this experiment showed that survival rate in ALA metabolites pre-treated mice were 40% and 10% extended than LPS alone challenged mice (Fig. 7A).

Since ALA metabolites showed a decrease in mortality rate in mice with endotoxin-mediated septic shock, the study was further extended to mice with sepsis induced by polymicrobial infection in a CLP mouse model. Results showed upregulation of iNOS and NLRP3 expression in CLP mice and their down-regulation in mice pre-treated with ALA metabolites (Fig. 7B). The levels of IL-1β and IL-10 were increased in liver tissue and serum of CLP mice. However, the level of pro-inflammatory cytokine-IL-1β was decreased while the level of anti-inflammatory cytokine-IL-10 was further increased in mice treated with ALA metabolites (Fig. 7C).

## Discussion

The ratio of ω-6 PUFAs: ω-3 PUFAs in cell membranes reflects the physiological status of the tissues and regulates the inflammatory response to a variety of external or internal stimuli and thus maintains cellular homeostasis^7^,^8^. ω-3 PUFAs are known to exhibit anti-inflammatory effects and reduce the oxidative stress in the cells^7^,^47^. The findings of the present study also support this concept wherein ALA, ω-3 PUFA, and its metabolites, 13-(S)-HPOTrE and 13-(S)-HOTrE, significantly reduced the production of NO and ROS, as well as inflammatory cytokines, in LPS stimulated RAW 264.7 cells and in mouse peritoneal macrophages. Further studies on *in vivo* sepsis model and CLP mice model demonstrated the protective role of 13-(S)-HPOTrE and 13-(S)-HOTrE as evidenced by a decrease in the mortality rate and by the maintenance of anti-inflammatory properties, in ALA metabolites treated BALB/c mice.

From previous studies, it has been established that ω-3 PUFAs exert their anti-inflammatory effects through several mechanisms, one of which being NF-κB signaling^7^,^47^. ω-3 PUFAs prevent the phosphorylation and degradation of Iκ-Bα protein by proteasome complex, thus inhibiting NF-κB signaling pathways^48^. The present study shows that 15-LOX metabolites of ALA [13-(S)-HPOTrE and 13-(S)-HOTrE] inhibit the translocation of NF-κB to the nucleus, along with the reduction of NO and ROS in LPS stimulated RAW 264.7 cells and peritoneal mouse macrophages. ALA metabolites also inhibited the activation of NLRP3 inflammasome as well as downstream signaling molecules such as caspase-1 and IL-1β at both transcriptional as well as translational levels. Interestingly, these effects of ALA metabolites on NLRP3 inflammasome were blocked by the PPAR-γ antagonist-GW9662. These studies comprehensively suggest that inactivation of LPS induced NLRP3 inflammasome by 13-(S)-HPOTrE or 13-(S)-HOTrE in RAW 264.7 cells and peritoneal macrophages is dependent on PPAR-γ. Indeed a number of studies have shown that several PUFA metabolites are ligands of PPAR-γ^19^, further supporting such a possibility.

Recent studies have established that the inducible isoform of COX, COX-2, has dual roles, pro-and anti-inflammatory, depending on the downstream metabolites generated^13^,^49^. In the early stages, it is shown that COX-2 induction is mainly associated with the generation of pro-inflammatory prostaglandins, whereas at later stages the downstream metabolite is PGD_2_, a ligand for PPAR-γ^17^, which has been observed to possess anti-inflammatory roles^50^. In the present study, an increase in the expression of COX-2 and its downstream metabolites (PGE_2_ and PGD_2_) was observed in RAW 264.7 cells pre-treated with ALA metabolites and then stimulated with LPS. Here, COX-2 may be playing an anti-inflammatory role by shifting PGE_2_ dominated prostaglandins in LPS stimulated macrophages to PGD_2_ dominated prostaglandins upon treatment with ALA metabolites. It has been reported that PGD_2_ derivative PGJ_2_ and their metabolites ^12^∆ PGJ_2_ and 15d-PGJ_2_, often derived from induced COX-2, exhibit their anti-inflammatory effects in various *in vivo* systems^20^,^15^,^51^. In the present study, the induced expression of COX-2 and increased formation of PGD_2_ upon treatment with ALA metabolites in LPS stimulated macrophages may be mediating the anti-inflammatory effects through its downstream metabolite, 15d-PGJ_2_ a natural ligand for PPAR-γ^52^. This hypothesis is supported by the observation that PPAR-γ is maintaining the anti-inflammatory state, by inhibiting inflammasome and IL-1β, via induced COX-2 generated downstream metabolites, and when PPAR-γ is inhibited, it is failing to inactivate the inflammasome. The reversed effects of ALA metabolites on inflammasome and caspase-1, when co-incubated with the PPAR-γ antagonist GW9662 support such a possibility.

Macrophage differentiation, their subtypes and function depend on its surrounding environment. After differentiation, macrophages polarized into two subtypes, M1 and M2 with characteristic phenotypes and exhibit different behaviours^53^. While, the activation of M1 macrophage is associated with increased expression of iNOS^54^ and increased level of pro-inflammatory cytokines such as TNF-α, IL-1β and IL-6^55^, the activation of M2 macrophages is associated with increased level of anti-inflammatory cytokines-IL-10 and scavenger receptor A^55^. LPS stimulated pro-inflammatory state is associated with M1 subtype macrophage, on the other hand, PPAR-γ activation and increased PGE_2_^56^,^57^ is associated with shifting of M1 macrophages into M2 macrophages subtypes^58^,^59^. In the present study, ALA metabolites activated PPAR-γ, and also increased PGE_2_ level that might be responsible for shifting of M1 to M2 macrophages subtypes and thereby mediating anti-inflammatory effects. Furthermore, an increased cytokine IL-10 as well as decreased expression of iNOS and TNF-α which are associated to M2 subtype macrophages further strengthen such possibility.

Apoptosis is the major mechanism for safe clearance of PMNLs at inflammatory sites and thus limit pro-inflammatory signals and promote resolution of inflammation rather than persistence of tissue damage^60^. In the present study, 13-(S)-HPOTrE showed a dose-dependent increase in apoptosis in LPS activated RAW 264.7 cells and it is previously reported that activation of PPAR-γ induces apoptosis in differentiated naive macrophages^61^. Therefore, it is possible that the apoptotic effects of 13-(S)-HPOTrE might be mediated through PPAR-γ dependent pathway. There is a correlation between apoptosis and regulation of autophagy. It is reported that apoptosis inhibits beclin-1 mediated autophagy^43^. It supports the present findings on immunofluorescence microscopy with LC3, in which pre-treatment of ALA metabolites remarkably diminished autophagy in LPS challenged RAW 264.7 cells. Furthermore, in immunoblot analysis, a decrease in beclin-1 expression was observed in ALA metabolites pre-treated and challenged with LPS. From these studies, it can be concluded that these ALA metabolites inhibit beclin-1 mediated autophagy through induction of apoptosis.

Sepsis is a critical state of the body because of systemic immune response induced by pathogens, microbes or endotoxins, also called systemic inflammatory response syndrome^62^. In endotoxin-induced septic shock model, mice pre-treated with ALA metabolites showed a significant decrease in mortality rate. Moreover, in another *in vivo* study, in CLP mouse model, mice treated with these ALA metabolites showed a decrease in expression of NLRP3. It also showed a reduction in IL-1β levels while elevation in IL-10 cytokine level as well. Thus the *in vitro* anti-inflammatory effects of 13-(S)-HPOTrE and 13-(S)-HOTrE observed in RAW 264.7 cells and peritoneal macrophages also have been demonstrated *in vivo* as evidenced by the extension in the survival of mice in endotoxin-induced septic shock in toxemia model and inactivated inflammasome in polymicrobial induced sepsis in CLP model.

In the present study, the hydroperoxy [13(S)-HPOTrE] and hydroxy [13(S)-HOTrE] metabolites of ALA showed anti-inflammatory effects, though the hydroperoxy metabolite being more potent. As the hydroperoxy metabolite [13(S)-HPOTrE] is getting converted to the corresponding hydroxy [13(S)-HOTrE] metabolite with increasing period of time because of the reducing environment created by serum in culture medium, the effects of 13(S)-HPOTrE observed in the present study may be mediated by both the hydroperoxy and hydroxy metabolites of ALA.

The anti-inflammatory properties of ALA (ω-3 PUFA) observed in the present study thus are quite contrasting to those of AA (ω-6 PUFA) metabolites, wherein the hydroperoxy (15-(S)-HPETE) and hydroxy (15-(S)-HETE) metabolites of AA showed contrasting effects on angiogenesis, which is critically associated with inflammation^27^,^63^,^64^. While 15(S)-HPETE showed anti-angiogenic effects^27^, the 15(S)-HETE induced angiogenesis^64^ in adipose tissue. Also, such differential effects of the hydroperoxy and hydroxy metabolites of 15-LOX on angiogenesis were observed in human umbilical vein endothelial cells (HUVECs)^63^. Similar differential effects of hydroperoxy and hydroxy metabolites of 15-LOX with AA as the substrate were reported on chronic myeloid leukemia cell line^26^. Interestingly, in the present study, the hydroperoxy and hydroxy metabolites of ALA showed anti-inflammatory effects, however, the hydroperoxy metabolite being more potent than the hydroxy metabolite. These findings, thus strongly advocate the beneficial effects of ω-3 PUFAs in counteracting various inflammatory disorders, which are mainly mediated by the metabolites of ω-6 PUFAs.

In summary, ALA metabolites [13-(S)-HPOTrE and 13-(S)-HOTrE] exhibit anti-inflammatory effects in LPS challenged RAW 264.7 cells and mouse peritoneal macrophages by down-regulating LPS induced pro-inflammatory markers and by inactivating the NLRP3 inflammasome complex. Additionally, the anti-inflammatory effects of ALA metabolites were mediated by the induction of apoptosis and inhibition of autophagy in the LPS challenged macrophages. These anti-inflammatory effects of ALA metabolites appear to be mediated by the inactivation of NLRP3 inflammasome complex and decrease in pro-inflammatory cytokines/enzymes along with a simultaneous increase in anti-inflammatory cytokines. The present study also demonstrates the protective effects of ALA metabolites *in vivo* as evidenced by the extended survival of BALB/c mice in LPS induced septic shock.

## Materials and Methods

### Chemicals and Reagents

Culture medium DMEM, penicillin, streptomycin, PBS, Trypsin-EDTA and fetal bovine serum (FBS) were purchased from Gibco. MTT, 2′-7′-Dichlorodihydrofluorescein diacetate (DCFH-DA), LPS (lipopolysaccharide from *E. coli, 0127:B8)*, Griess reagent, Dexamethasone (Dex) and GW9662 were purchased from Sigma-Aldrich, USA. Anti-Beclin-1, anti-Caspase-1, anti-NF-κB and anti-TNF-α antibodies were purchased from Abcam (MA, USA). Anti-LC-3 antibody was purchased from Cell Signaling while Anti-COX-2 antibody was obtained from Santa Cruz Biotechnology Inc. (Texas, USA). Anti-iNOS antibody was purchased from Thermo Fisher Scientific (MA, USA). Enzyme immunoassay kit for IL-1β, IL-10 and PGE_2_ were purchased from R&D system, Inc. (MN, USA) whereas PGD_2_ assay kit was purchased from Cayman Chemical Company (Ann Arbor, MI, USA). AA, LA, ALA and DHA were procured from Nu-Chek-Prep (MN, USA). The 15-LOX metabolites of ALA, 13-(S)-HPOTrE and 13-(S)-HOTrE, were generated (Supplementary Figure S6) essentially employing the methods described earlier for generation of 5-LOX metabolites^65^,^66^,^67^.

### Cell culture and Treatments

The mouse macrophage cell line, RAW 264.7 was obtained from NCCS (Pune, India) grown and maintained in DMEM supplemented with 10% heat-inactivated fetal bovine serum (FBS), 100 IU/ml penicillin and 100 μg/ml streptomycin in a humidified atmosphere with 5% CO_2_ at 37 °C. Subculturing of cells was done twice a week. Fresh culture medium was used before each treatment. Cells were pre-incubated with different concentrations (1, 50 and 100 μM) of ALA metabolites, 13-(S)-HPOTrE and 13-(S)-HOTrE or with Dexamethasone (10 μM) for 3 h then challenged with LPS (100 ng/ml) for different time points as mentioned in experiments. The anti-inflammatory drug, dexamethasone was used as positive control. For inhibitor study, cells were pre-incubated with 10 μM GW9662 (2-chloro-5-nitrobenzanilide), for specific time period as mentioned in experiments. For analysis of the inflammasomes and IL-1β measurements, RAW 264.7 cells and peritoneal macrophages were incubated with 1 mM ATP for 30 min prior to end time points.

### Animal

BALB/c, male mice, 4-week old weighing 20–25 g were purchased from Centre for Cellular and Molecular Biology, Hyderabad, India. Mice were housed at constant room temperature (23 ± 1 °C) and allowed to water and food *ad libitum* in 12 h dark/light cycle. The mice were kept at least 1 week in animal house before performing any experiment. The mice used in this study were handled carefully and according to the *Guide for the care and use of Laboratory animals* published by NIH (National Institute of Health). The experimental protocol was approved by Institutional Animal Ethics Committee (IAEC), University of Hyderabad, India.

### LPS Induced Septic Shock

BALB/c male mice (20 to 25 g) were divided into 4 different groups (10 mice in each group), control, LPS, LPS+13-(S)-HPOTrE and LPS+13-(S)-HOTrE. LPS was dissolved in saline (0.9% NaCl) and injected (30 mg/kg body weight) intraperitoneal (i.p.) in all mice except control. In control mice, 0.9% saline was injected. ALA metabolites were dissolved in saline having Tween 80 (0.9% NaCl + 0.5% Tween 80). Two i.p. doses of 13-(S)-HPOTrE and 13-(S)-HOTrE (0.1 mg/kg body weight, 2.5 μg/mice) metabolites were injected, first at 1 h prior to LPS and second, soon after LPS administration. 250 μl saline with Tween 80 (vehicle control) was injected in control and LPS treated groups. All mice were kept in normal conditions with an extra vigilance. Mice were monitored initially at 2 h intervals for 12 h then for 4 days.

### Cecal Ligation and Puncture (CLP) model

Cecal Ligation and Puncture was performed in BALB/c male mice (20 to 25 g). Mice were divided into 4 groups (5 mice/group), Sham, CLP, CLP+13-(S)-HPOTrE and CLP+13-(S)-HOTrE. The mice were anesthetized with 60 μg/g ketamine and 10 μg/g xylazine. After cleaning and disinfecting the lower abdomen by iodine solution to prevent infection, a 1.5 to 2 cm incision was made through linea alba. Then, the cecum was spotted and ligated with disinfected 3–0 silk suture and perforated twice using a 22-gauge needle. Wound strength was ensured by squeezing out a small amount of stool. Then the cecum was replaced into the abdomen, and the incision was closed properly. In Sham, mice undergone surgery but ligation and puncture were not performed. Tramadol hydrochloride (20 μg/g body wt.) was injected i.p. with 1 ml warm saline in all mice. In ALA metabolites treated mice, two i.p. doses of 13-(S)-HPOTrE and 13-(S)-HOTrE (0.1 mg/kg body weight, 2.5 μg/mice) metabolites were given, first at 1 h prior to CLP and second, soon after CLP surgery. Finally, all mice were kept at normal conditions with an extra vigilance. The mice were sacrificed after 24 h of CLP surgery then blood, peritoneal fluid and liver were collected.

### Isolation of Primary Peritoneal Macrophages

Primary peritoneal macrophages were isolated from thioglycolate-elicited BALB/c male mice as described by Xia Zhang *et al.*^68^. Briefly, in BALB/c male mice, 3% thioglycolate broth (1 ml) was injected through i.p. After 3 days, mice were euthanized by cervical dislocation. 5 ml ice-cold DMEM was injected into the peritoneal cavity. The abdomen was finger tapped 4–5 times and peritoneal lavage was aspirated and collected in a cold sterile centrifuge tube. Further, centrifugation was done at 1500 rpm for 5 min, pellets were resuspended in DMEM and cell counting was done by haemocytometer. Appropriate number of cells were grown and maintained in DMEM, macrophages enrichment was done by washing and removing unattached cells from culture plate, further grown as described above for RAW 264.7 cells, and treated with 13-(S)-HPOTrE and 13-(S)-HOTrE as mentioned in experiments for RAW 264.7 cells.

### Cytotoxicity Assay

Cytotoxicity of PUFAs and their metabolites were assayed by MTT assay. To perform this assay, RAW 264.7 cells were seeded in 96 well plates at a density of 5 × 10^4^ cells/well then treated with ω-6 PUFAs (AA and LA), ω-3 PUFAs (ALA and DHA) and 15-LOX metabolites of ALA [13-(S)-HPOTrE and 13-(S)-HOTrE] at various concentrations (1, 50, 100 and 200 μM) for different time points. Cell viability was measured colorimetrically by MTT (3-(4,5-dimethylthiazol-2-yl)-2,5-diphenyl tetrazolium bromide) assay as described by Mossman^69^.

### Nitrite Estimation

RAW 264.7 cells were seeded in 6 well plates for 12 h then pre-incubated with different concentrations (1, 50 and 100 μM ) of ALA metabolites [13-(S)-HPOTrE and 13-(S)-HOTrE], or dexamethasone for 3 h then stimulated with or without LPS (100 ng/ml) for the next 24 h. At the end of the time point, stable nitrite level in the culture supernatant was measured by Griess Reagent. For this, 50 μl of cell culture medium was mixed with 50 μl of Griess reagent and incubated at room temperature for 10 min in the dark. Then absorbance was measured at 540 nm using multi-mode plate reader (BioTek, SynergyMx). Nitrite level in samples was determined by using the standard curve of sodium nitrite. Culture medium was taken as blank for all experiments.

### Estimation of IL-1β, IL-10, PGD_2_ and PGE_2_

Levels of IL-1β, IL-10, PGE_2_ and PGD_2_ in culture medium were measured by ELISA kit according to manufacturer’s protocol. Their levels in culture medium were determined by using standard curves of respective cytokine or prostaglandin standards provided along with the kit.

### Measurement of Reactive Oxygen Species (ROS)

RAW 264.7 cells were seeded in 6 well plates at a density of 2 × 10^5^ cells/well then pre-incubated with different concentrations (1, 50 and 100 μM ) of ALA metabolites [13-(S)-HPOTrE and 13-(S)-HOTrE] and/or N-Acetyl Cysteine (NAC, 5 mM), for 3 h. Then cells were further stimulated with or without LPS (1 μg/ml) for next 16 h. After that cells from each well were harvested and washed twice with PBS and further incubated with 10 mM DCFH-DA for 30 min in dark at 37 °C and then two times washed with PBS. About 10,000 cells per sample were taken for analysis. The measurement was performed on a Flow Cytometer and data analysis was done by Cell Quest software (FACS Calibur, Becton Dickinson, CA, USA) with excitation of DCF at λ488 nm and emission at λ525 nm. Primary macrophages isolated from peritoneal cavity were seeded in 12 well plate and grown as mentioned earlier. Then pre-treated with 13-(S)-HPOTrE and 13-(S)-HOTrE at 100 nM for 3 h. Then stimulated with LPS and ROS was measured as described above.

### Immunoblot Analysis

RAW 264.7 cells seeded in 100 mm plates at a density of 5 × 10^5^ cells/well and then after 12 h pre-incubated with different concentrations (1, 50 and 100 μM) of either ALA metabolites or dexamethasone (10 μM) for 3 h. For Immunoblot analysis of primary cells, mouse peritoneal macrophages were isolated and seeded in 60 mm plates. Then pre-treated with lower doses (100 nM and 400 nM) of ALA metabolites for 3 h. Cells were then stimulated with or without LPS (100 ng/ml) for next 24 h. Cells were harvested at the end of time point, pelleted down and stored at −80 °C for further use. Cell pellets were lysed in RIPA buffer containing 1x protease inhibitor at 4 °C. Protein estimation was done by Bradford method using BSA as standard. An equal amount of protein was loaded and separated on 8–15% SDS-PAGE and transferred to nitrocellulose membrane. Then membranes were blocked with 5% (w/v) fat-free dry milk in TBST for 1 h at room temperature followed by washing three times with TBST. Membranes were incubated with primary antibodies (0.5–1.0 μg/ml) for 12 h at 4 °C on a shaker incubator with gentle shaking followed by thrice washing with TBST. The membranes were then incubated with respective secondary antibodies conjugated with HRP. Signals were then detected with western lightning plus ECL kit (PerkinElmer) and captured on Kodak Imaging System (KODAK Image station 4000 mm Pro).

### Reverse Transcription Polymerase Chain Reaction (RT-PCR)

RAW 264.7 cells were seeded in 6 well culture plate. At 60% confluency, cells were pre-incubated with different concentrations of either ALA metabolites (1, 50 and 100 μM) or dexamethasone (10 μM) for 3 h and then stimulated with LPS (100 ng/ml) for 24 h. Total cellular RNA was extracted from each well using TRIzol^®^ Reagent according to the manufacturer’s instructions (Invitrogen Bio Services, India, Pvt. Ltd). RNA quantification was performed by nanodrop (NanoDrop 2000TM, Thermo Scientific, DE, USA). cDNA of each sample was prepared by using 2 μg of RNA, 1 μl MLV reverse transcriptase, 1 mM dNTP and 1 μl oligo dT according to manufacturer’s standardized protocol (Promega Corporation, WI, USA). PCR analyses were performed on aliquots of the cDNA prepared to detect iNOS, TNF-α, NLRP3, Caspase-1, IL-1β, IL-18 and GAPDH. The reactions were carried out in a volume of 20 μl containing (final concentration) 1 U Taq DNA polymerase, 0.2 mM dNTP, 100 pM of forward primers and reverse primers (Supplementary Table S1), and 10 μl reaction buffer. After amplification, PCR products were electrophoresed on 1% agarose gels and visualized by ethidium bromide staining on UV irradiation (BIO-RAD, Universal hood II).

### Analysis of the inflammasome, autophagy and NF-κB translocation by confocal microscopy

RAW 264.7 cells were seeded in 6 well culture plates on sterile culture grade coverslips. When cells were 60% confluent, cells were pre-incubated with ALA metabolites (100 μM) and/or with GW9662, PPAR-γ inhibitor, for 3 h, then stimulated with LPS (100 ng/ml) for 24 h. For the study of inflammasome and autophagy, stimulation of cells with LPS was done for 24 h and for NF-κB translocation, it was done for 16 h. However, for study NF-κB translocation on peritoneal macrophages, cells were treated with ALA metabolites at lower concentration (100 nM) for 16 h. Fixation of cells was done by ice-cold 4% paraformaldehyde for 10 min at 4 °C followed by washing in PBS with 0.2% Tween 20. Permeabilization in cells was done by ice-cold acetone: methanol (1:3) for 15 min at room temperature followed by washing in PBS with 0.2% Tween 20. To avoid nonspecific binding, blocking was done by incubating cells in 5% FBS in PBST for 1 h at room temperature followed by twice washing in PBS with 0.2% Tween 20. Cells were then incubated with targeted primary antibody (1:100 dilution) in 3% BSA in PBST for 12 h at 4 °C followed by washing thrice in PBS with 0.2% tween 20. Cells were then incubated with the fluorescent conjugated respective secondary antibody (1:300 dilutions) in 3% BSA in PBST for 1 h at room temperature in the dark. Finally, washings were done and coverslips mounted on slides by anti-fade reagent with DAPI (ProLong Gold Antifade reagent, Invitrogen, USA). Image analysis was done on a confocal microscope (Zeiss LSM700, USA).

### Analysis of Apoptosis by FACS

RAW 264.7 cells were seeded in 6 well cultures plate. When cells were 60% confluent, cells were pre-incubated with different concentrations of either ALA metabolites (1, 50 and 100 μM) and/or doxorubicin (10 μM) for 3 h, then stimulated with LPS (100 ng/ml) for 24 h. Staining of cells was done by FITC Annexin V Apoptosis Detection Kit (BD Biosciences, CA, USA), according to manufacturer’s standardized protocol. Samples were run on Flow Cytometer (BD, LSR Fortessa, CA, USA) and data analyses were done on BD FACS Diva™ software.

### Statistical Analyses

All the experiments were performed in triplicates and the values represented as mean ± SD. Data were correlated, analyzed and p-values were obtained using student’s t-test. The p-value < 0.05 was considered as statistically significant.

## Additional Information

**How to cite this article**: Kumar, N. *et al.* 15-Lipoxygenase metabolites of α-linolenic acid, [13-(S)-HPOTrE and 13-(S)-HOTrE], mediate anti-inflammatory effects by inactivating NLRP3 inflammasome. *Sci. Rep.* **6**, 31649; doi: 10.1038/srep31649 (2016).

## Supplementary Material

Supplementary Information
