# Supplementary material for: 15-Lipoxygenase metabolites of α-linolenic acid, [13-(S)-HPOTrE and 13-(S)-HOTrE], mediate anti-inflammatory effects by inactivating NLRP3 inflammasome
Source: Sci Rep. 2016 Aug 18;6:31649. doi: 10.1038/srep31649 (PMC4989172; doi:10.1038/srep31649)
Supplement: Supplementary Information [file srep31649-s1.pdf]

15-Lipoxygenase metabolites of α-linolenic acid, [13-(S)-HPOTrE and 13-(S)-HOTrE], mediate anti-inflammatory effects by inactivating NLRP3 inflammasome

Naresh Kumar[1], Geetika Gupta[1], Kotha Anilkumar[2], Naireen Fatima[1], Roy Karnati[1], Gorla Venkateswara Reddy[1], Priyanka Voori Giri[1] and Pallu Reddanna[1*]

[1]School of Life Sciences, University of Hyderabad, Hyderabad 500046, India
[2]National Institute of Animal Biotechnology, Hyderabad 500049, India

*Corresponding Author (E-mail: prsl@uohyd.ernet.in)

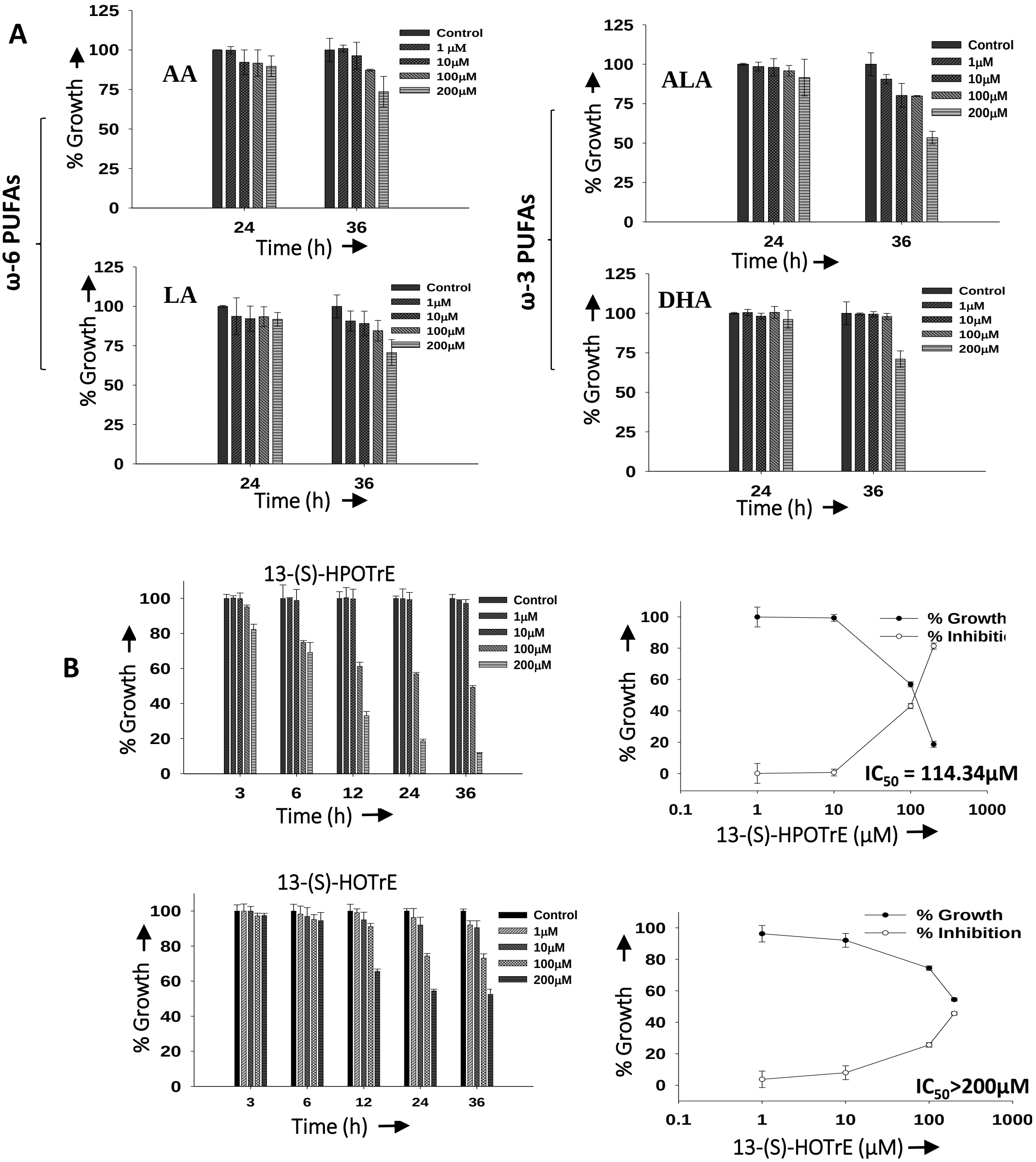

Supplementary Figure S1| Effects of PUFAs and their 15 LOX metabolites on viability of mouse macrophage cell line, RAW 264.7 cells. In MTT assay, Cells were treated with different concentrations (1, 10, 100 and 200 µM) of **A)** PUFAs (AA, LA, ALA and DHA) and **B)** 15 LOX metabolites of ALA [13-(S)-HPOTrE and 13-(S)-HOTrE] for different time points (as mentioned in above graphs). The percent cell growth following treatment was calculated, in comparison with untreated control cells.

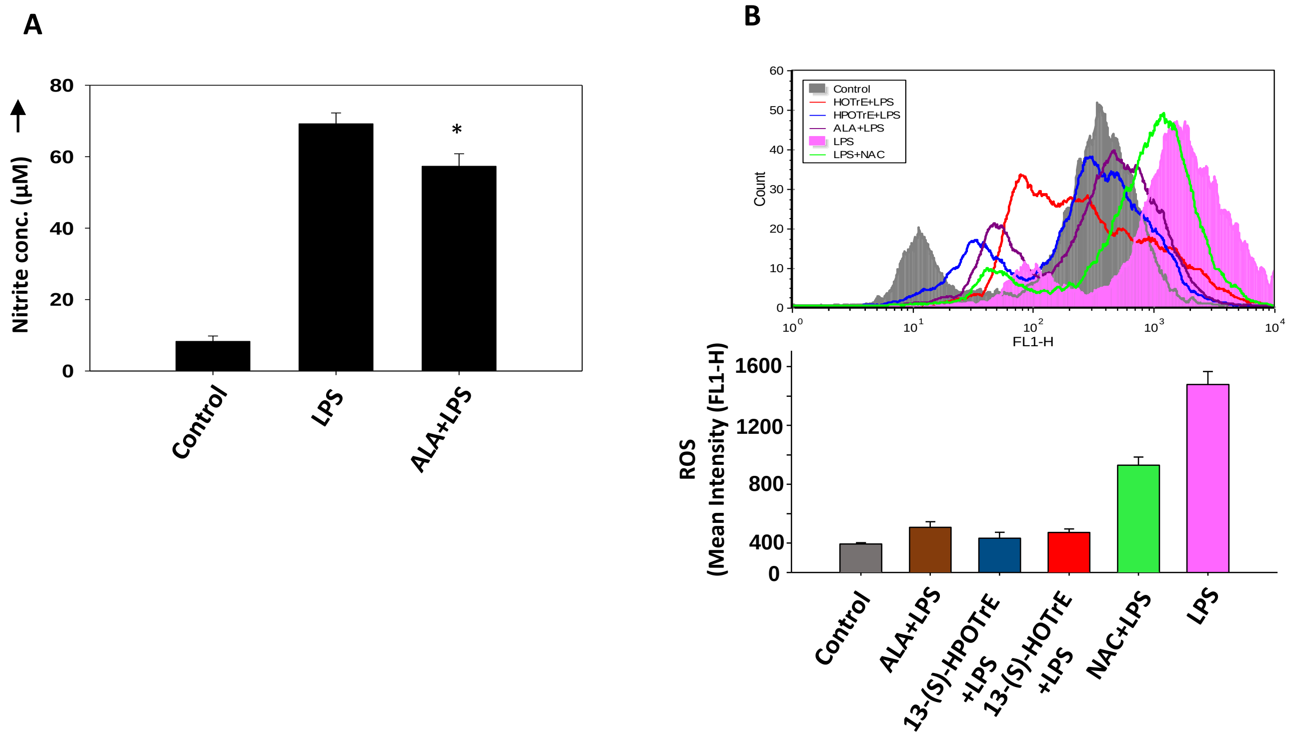

Supplementary Figure S2| Effects of 13-(S)-HPOTrE and 13-(S)-HOTrE on generation of NO and ROS in RAW 264.7 cells. A) Nitrite levels in culture medium of RAW 264.7 cells pre-incubated with ALA (100 µM) for 3 h and further stimulated with or without LPS (100 ng/ml) for the next 24 h. ALA showed significant reduction in NO level as compared to LPS. The values represent mean ±SD of three independent experiments. * indicates significance ($p<0.05$) compared to LPS alone treated cells. **B)** Intracellular ROS level in cells following pre-incubation with ALA (100 µM), 13-(S)-HPOTrE (100 µM) and 13-(S)-HOTrE (100 µM) for 3 h then stimulation with LPS for 16 h. 13-(S)-HPOTrE reduced ROS level more efficiently compared to 13-(S)-HOTrE and ALA. N-Acetyl Cysteine (NAC, 5mM), a ROS inhibitor was used as positive control.

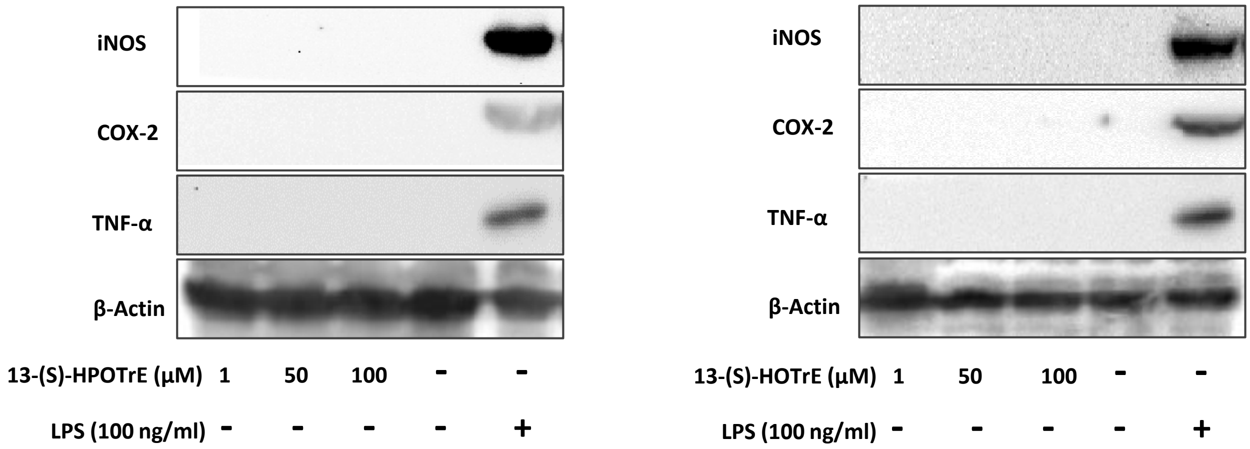

Supplementary Figure S3| Effects of ALA metabolites on the expression of iNOS, COX 2 and TNF-α in unstimulated RAW 264.7 cells. Immunoblot analysis showing the expression of iNOS, COX-2 and TNF-α following treatment with 13-(S)-HPOTrE and 13-(S)-HOTrE (1, 50 and 100 µM concentrations) for 24 h without LPS stimulation. β-Actin was used as an internal control. These are representative blots of the three independent experiments.

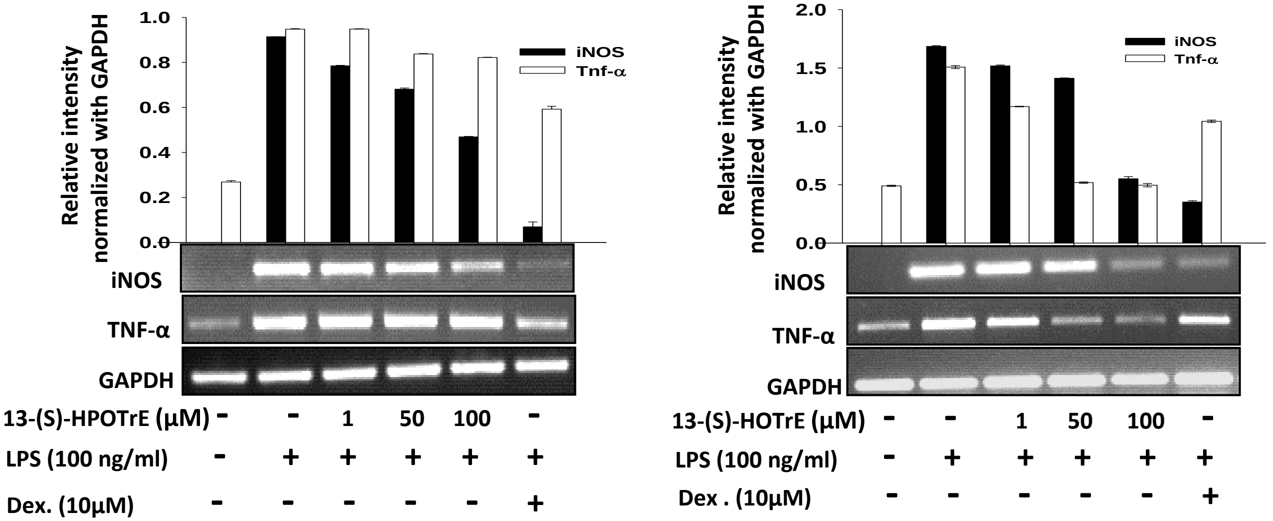

Supplementary Figure S4| Anti-inflammatory effect of ALA metabolites is mediated by inhibition of iNOS and TNF-α in RAW 264.7 cells. RT-PCR analysis of transcripts of iNOS and TNF-α in RAW 264.7 cells pre-treated with ALA metabolites for 3 h then stimulated with LPS (100ng/ml) for 24 h. GAPDH was used as an internal control. Dexamethasone was used as positive control. These are representative gel images of the three independent experiments. The relative band intensities were measured by quantative scanning densitometry (Image J).

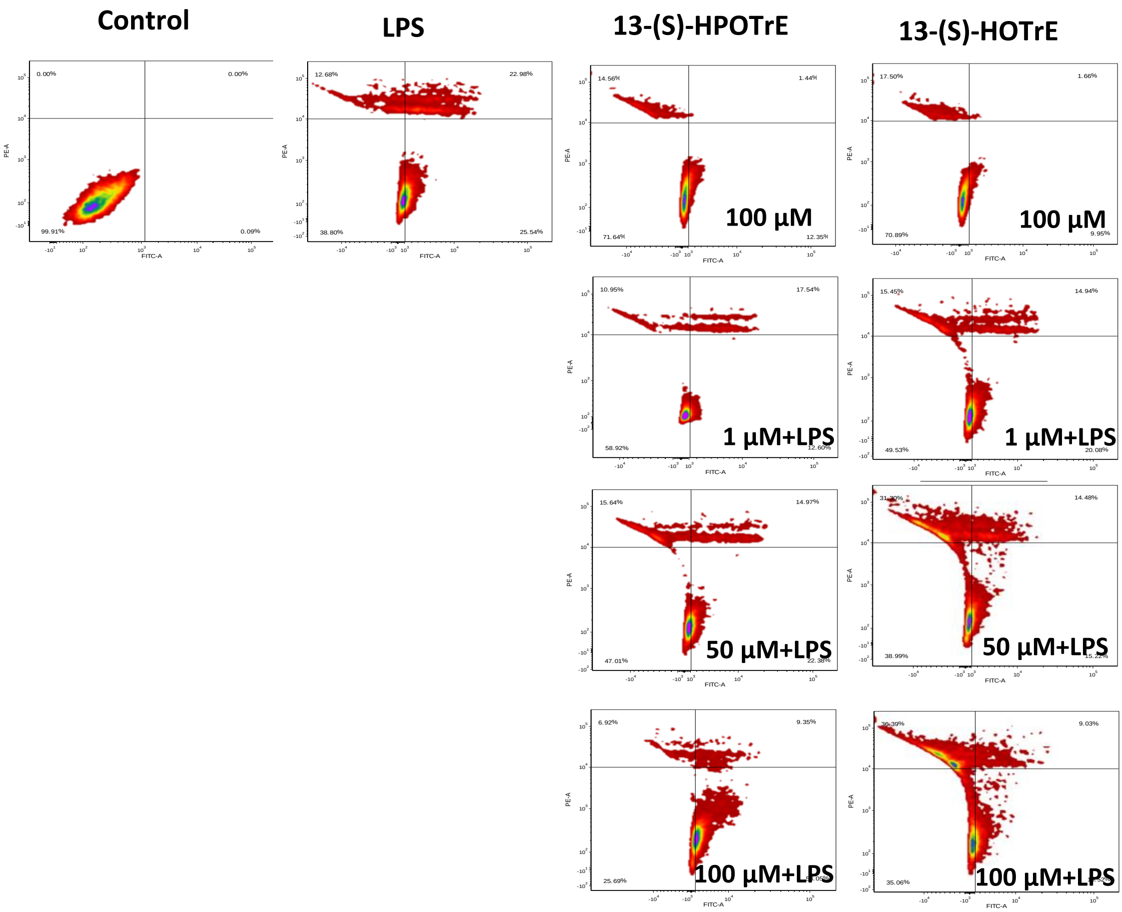

Supplementary Figure S5| Effects of 13-(S)-HPOTrE and 13-(S)-HOTrE on apoptosis in RAW 264.7 cells, during LPS induced inflammation. Apoptosis was assayed by Propidium Iodide and FITC conjugated Annexin V at 24 h by flowcytometric Analysis. The apoptosis level was calculated as % of AnnexinV^{+} PI^{-} cells in density plot distribution. The dead cells were gated and analysis was performed only on live cells. The image shown here is representative of three independent experiments.

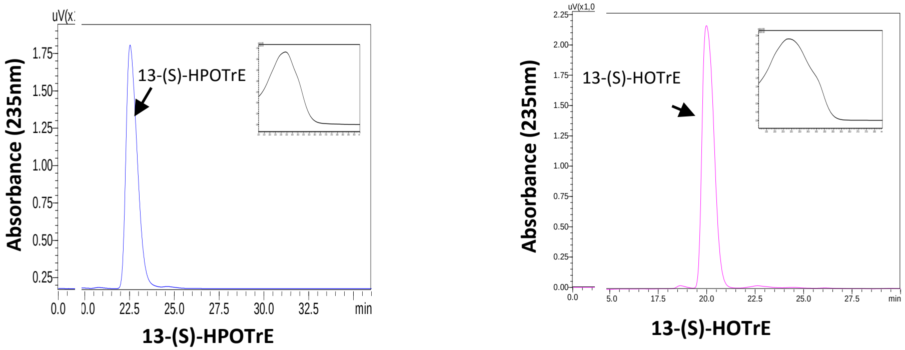

Supplementary Figure S6|Hydroperoxy and Hydroxy Metabolites of ALA [13-(S)-HPOTrE and 13-(S)-HOTrE] were Synthesized by 15-Lipoxygenase. Linseed 15-Lipoxygenase was employed for synthesis of hydroperoxy (13-(S)-HPOTrE) and hydroxy metabolites (13-(S)-HOTrE) of ALA. 15-LOX was incubated with ALA in 100 mM Tris-Cl buffer, pH 9.6 for 3 min. The products formed were extracted with equal volumes of hexane: diethyl ether (1:1). The organic solvent was evaporated and dried products were then dissolved in HPLC mobile phase consisting of hexane: propane-2-ol: acetic acid in 1000:15:1 ratio for purification of these metabolites on HPLC. For biosynthesis of corresponding hydroxides, these dried hydroperoxy products were reduced with sodium borohydride ($NaBH_4$).

The metabolites were separated on semi preparative straight phase HPLC (Shimadzu LC 20AP, Shimadzu LC solution), using Enable Silica column (250x20mm) on the solvent system mentioned above at a flow rate of 6 ml/min. The samples were monitored at 235 nm and peaks were analysed on UV/VIS Perkin Elmer lambda35 spectrophotometer. The solvent evaporated and metabolites were stored in 100% ethanol in inert environment at -80°C. The peaks with characteristic 235 nm were collected and identified by Mass spectral analysis on LC-MS/MS (LC: Agilent Technologies, 1200 series, Hystar Version 3.2, MS/MS: Bruker Daltonics, micrOTOFQ, micrOTOF control version 2.2).

Table 1:	List of Primers used for RT-PCR
**mRNA**	**Sequences of Primers**
NLRP3	Forward Primer 5’ CTT CCA GAC TGG TGA ACT GCTG 3’,<br>Reverse Primer 5’ TCT CCA AGG GCA TTG CTT CG 3’,
Caspase-1	Forward Primer 5’ GAG CTT CAA TCA GCT CCA TCA G 3’<br>Reverse Primer 5’ AAT GTC CCG GGA AGA GGT AGA 3’,
IL-1β	Forward Primer 5’ AGC TTC CTT GTG CAA GTG TC 3’,<br>Reverse Primer 5’ AAG GGA GCT CCT TAA CAT GCC 3’,
IL-18	Forward Primer 5’ TTC CTC TTG GCC CAG GAA CAA TG 3’<br>Reverse Primer 5’ CAA GGC GCA TGT GTG CTA ATC 3’
GAPDH	Forward Primer 5’ AGG TCA TCC CAG AGC TGA ACG 3’<br>Reverse Primer 5’ CAC CCT GTT GCT GTA GCC GTA T 3’.
iNOS	Forward primer: 5’ CCG CAT GAG CTT GGT GTT TG 3’<br>Reverse primer: 5’ AGT GCT TCA GTC AGG AGG TTG 3’
TNF-α	Forward Primer 5’ TGT AGC CCA CGT CGT AGC AAA 3’<br>Reverse Primer 5’ AGA GGC AAC CTG ACC ACT CT 3’,

Supplementary Table S1: List of Primers used for Semi-quantitative PCR

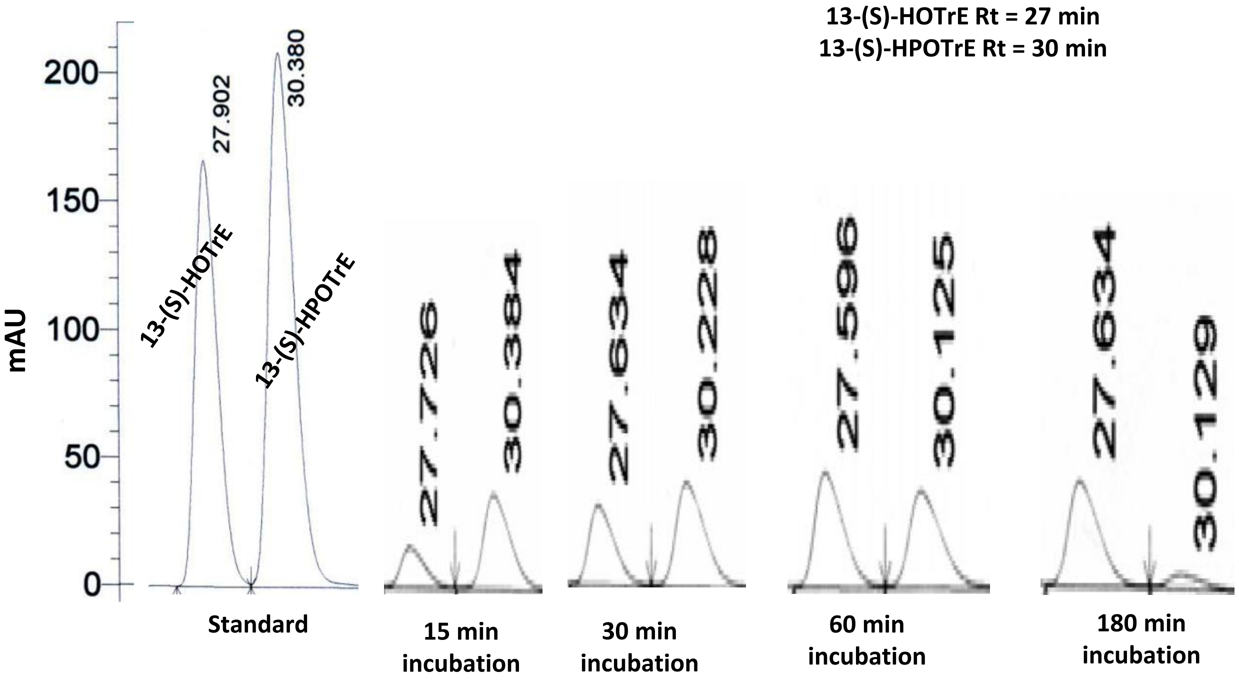

Supplementary Figure S7| Reducing environment of serum (FBS) reduced 15-LOX hydroperoxy metabolites of ALA (13-(S)-HPOTrE) into hydroxy metabolite (13-(S)-HOTrE) during incubation in RAW 264.7 cells. RAW 264.7 cells was grown in 10 % FBS and incubated with 10 µM 13-(S)-HPOTrE for 15, 30, 60 and 180 min. Culture medium was collected and reaction was arrested by adding 2N HCl at the end of time points. The products formed were extracted in hexane: diethyl ether (1:1) as described in supplementary figure S6. The metabolites were separated on analytical straight phase HPLC (Shimadzu LC 20AP, Shimadzu LC solution), using Silica column on hexane: propane-2-ol: acetic acid (1000:15:1) at 1 ml/min flow rate. The samples were monitored at 235 nm. Reduction of hydroperoxy metabolite (13-(S)-HPOTrE) into hydroxy metabolites (13-(S)-HOTrE), in complete medium (DMEM+10% FBS) was observed during incubation period and a significant amount of 13-(S)-HPOTrE was converted into 13-(S)-HOTrE at 180 min incubation time. Figure show cropped HPLC chromatograms for analysis purpose.
